# Specific targeting of adipose tissue metabolism is superior to caloric restriction in treating obesity-related HFpEF

**DOI:** 10.1186/s12933-025-02879-2

**Published:** 2025-07-30

**Authors:** Simon Sedej, Alina Stockner, Renate Schreiber, Abhinav Diwan, Rolf Breinbauer, Mahmoud Abdellatif

**Affiliations:** 1 Department of Cardiology, Medical University of Graz, Auenbruggerplatz 15, 8036 Graz, Austria; 2 BioTechMed Graz, Mozartgasse 12/II, 8010 Graz, Austria; 3 Faculty of Medicine, University of Maribor, Taborska ulica 8, 2000 Maribor, Slovenia; 4 Institute of Molecular Biosciences, University of Graz, Humboldtstrasse 50, 8010 Graz, Austria; 5 Division of Cardiology and Center for Cardiovascular Research, Washington University School of Medicine, St. Louis, MO USA; 6 John Cochran Veterans Affairs Medical Center, Washington University School of Medicine, St. Louis, MO USA; 7 Institute of Organic Chemistry, Graz University of Technology, Stremayrgasse 9/Z4, 8010 Graz, Austria

**Keywords:** Adipose tissue, Caloric restriction, HFpEF, Inflammation, Lipolysis, Obesity

## Abstract

Obesity is a modifiable major driver of heart failure with preserved ejection fraction (HFpEF), the most common and rapidly increasing form of heart failure. Current metabolic therapies, such as caloric restriction and incretin-based drugs, have shown promise in treating obesity-related HFpEF. However, these interventions neither specifically nor selectively improve adipose tissue metabolism, which is a key etiological factor in HFpEF that may offer a pathway to safer and more effective treatment strategies. Towards this end, we found that genetic inhibition of adipose triglyceride lipase (ATGL) specifically in adipocytes is sufficient to prevent the development of obesity-related HFpEF, and that pharmacological inhibition of ATGL using atglistatin effectively treats established disease. Atglistatin selectively inhibits ATGL in adipose tissue, but not in the heart, leading to superior reduction in adiposity and greater improvement in diastolic dysfunction compared to caloric restriction. These observations underscore the therapeutic potential of selectively targeting adipose tissue, independent of the effects of body weight loss. Mechanistically, atglistatin attenuates HFpEF-associated elevation of inflammatory cytokines, especially IL-1β levels in adipose tissue, more effectively than caloric restriction. In sum, these findings identify dysregulated adipose tissue metabolism as a causal factor and therapeutic target in maladaptive fat-heart crosstalk driving obesity-related HFpEF.

## Obesity as a key driver and modifiable risk factor for HFpEF pathogenesis

The prevalence of obesity has reached epidemic proportions globally, posing major challenges to public health. Obesity impacts multiple organ systems, yet approximately two-thirds of the excess mortality associated with obesity is attributed to cardiovascular disease [1]. A large body of preclinical and clinical evidence has emphasized the key mechanistic role of adiposity in the pathogenesis of age-related cardiac diseases [2], particularly heart failure with preserved ejection fraction (HFpEF) [3]. HFpEF is a multi-organ syndrome that is rapidly increasing in prevalence, largely due to population aging and obesity [4]. Despite the established links between obesity and clinically evident cardiometabolic HFpEF [5], obesity itself has been surprisingly underappreciated and sub-optimally managed compared to its associated metabolic complications and other modifiable cardiovascular risk factors, such as hypertension. Until recently, this shortcoming has limited the development of effective therapies for this predominant form of heart failure, underscoring the importance of understanding (i) how increased adiposity and its metabolic sequelae pathophysiologically contribute to cardiometabolic HFpEF, and (ii) how obesity and related metabolic complications can be targeted therapeutically to reinstate both adipocyte and cardiomyocyte health.

## Interventions targeting adipose tissue-heart crosstalk in HFpEF

Obesity and related metabolic dysfunction chronically elevates the levels of circulating fatty acids, initiating the development of multiple obesity-associated pathologies, including insulin resistance, diabetes, dyslipidaemia and chronic inflammation. These metabolic disturbances may directly impact the myocardium through proinflammatory cytokines and adipokines, leading to cardiac remodelling and dysfunction in HFpEF [6]. Therefore, targeting the unfavourable cardiometabolic profile through weight loss may represent a powerful treatment strategy to improve the outcomes in obese patients with HFpEF. In fact, modest weight loss caused by caloric restriction improves exercise intolerance, which is a cardinal feature of HFpEF, correlating with symptoms and reduced quality of life [7]. However, a modest weight loss may not be enough to improve cardiac structure and function as a significant reduction of > 10% in visceral adipose tissue is needed to achieve a sustained effect [8]. Furthermore, weight loss by lifestyle interventions is challenging to achieve and is rarely sustained for a longer period. Caloric restriction may also produce some negative effects, the underlying mechanisms of which are not yet fully understood and warrant further investigation, especially in humans [9]. Similarly, extreme measures such as bariatric surgery are not universally feasible and come with considerable risks and side effects. Therefore, a pharmacological treatment that is more effective and safer than caloric restriction or surgery to achieve long-term and sustainable weight loss is worth considering. In this context, clinical trials with sodium-glucose co-transporter 2 (SGLT2) inhibitors [10], and glucagon-like peptide-1 receptor analogs (GLP-1RAs) showed that pharmacological targeting of obesity and related whole-body metabolic derangements mitigates HFpEF [11]. These studies demonstrated that the extent of HFpEF improvement correlates with the degree of weight loss. However, although both caloric restriction and emerging HFpEF pharmacotherapies are powerful anti-obesity treatments that have proven multi-organ benefits in patients with HFpEF, they do not specifically or directly act on adipose tissue. Thus, directly targeting adipose tissue may provide to be a more selective and potentially safer treatment option. In line with this idea, adipose-centric interventions that target fatty acids mobilization within the adipose tissue (ie, lipolysis) could potentially interrupt the maladaptive fat-heart crosstalk, and might effectively and safely attenuate cardiac remodelling and dysfunction in HFpEF.

## Selective inhibition of lipolysis in adipose tissue treats obesity-related HFpEF

**Fig. 1:**
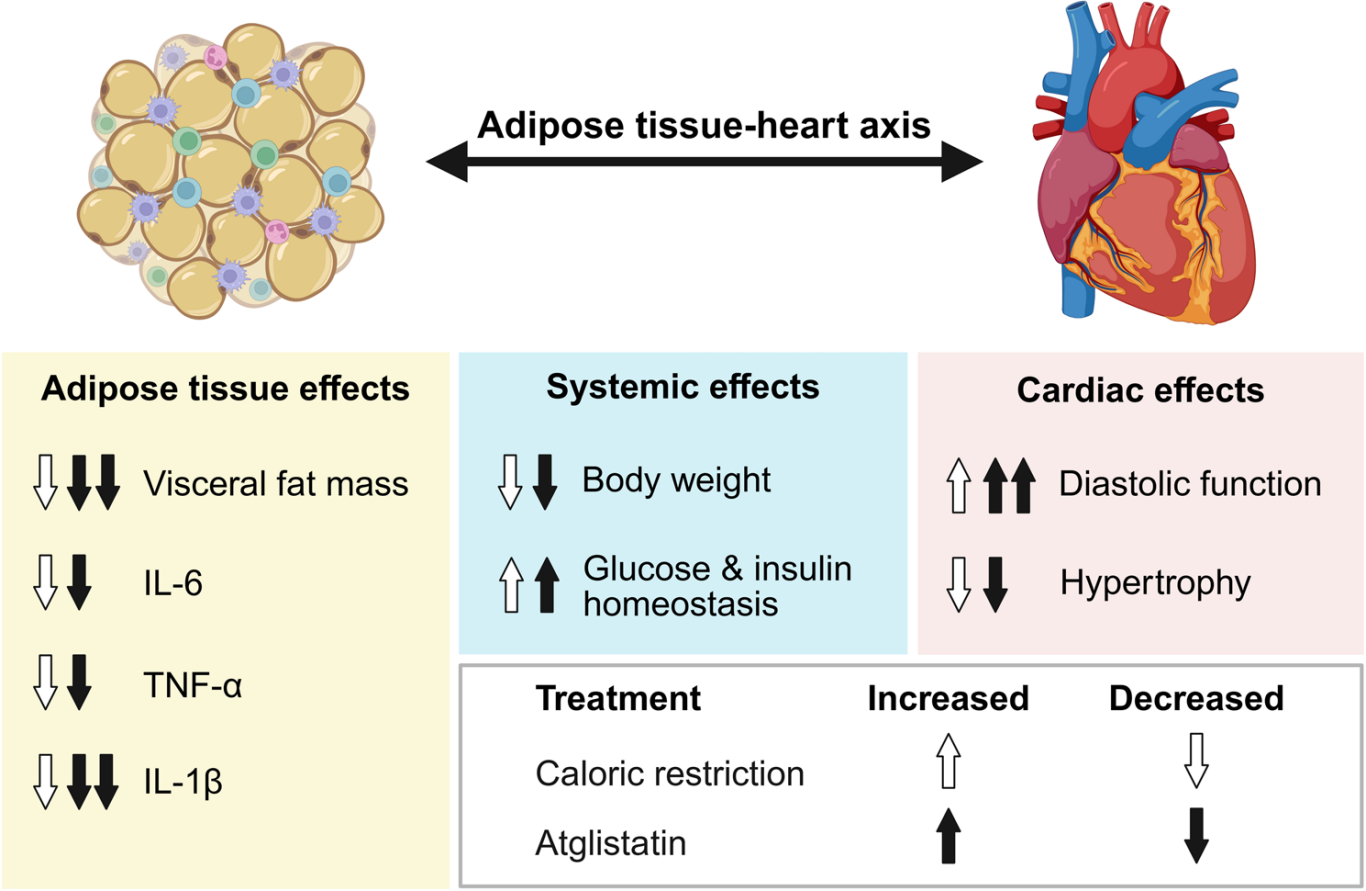
Atglistatin is superior to caloric restriction in treating obesity-related HFpEF. The illustration depicts a comparison of the molecular, cellular, and tissue-level cardiometabolic benefits induced by atglistatin and caloric restriction. White arrows indicate effects that are up- or downregulated in response to caloric restriction, whereas black arrows represent changes specifically induced by atglistatin. Atglistatin-induced effects that are superior to those observed under caloric restriction are emphasized by double arrows. Abbreviations: IL-6, interleukin-6; IL-1β, interleukin-1beta; TNF-α, tumor necrosis factor-alpha. Figure was created with BioRender.com licensed to the Medical University of Graz

We have recently demonstrated that specific targeting of lipolysis in adipose tissue through genetic deletion or pharmacological inhibition of the rate-limiting enzyme in triacylglycerol hydrolysis, adipose tissue triglyceride lipase (ATGL), is sufficient to protect from and even treat obesity-related HFpEF induced by the combination of a high-fat diet and the nitric oxide synthase inhibitor, L-NAME (ie, two-hit HFpEF model) [12]. Specifically, HFpEF mice with adipocyte-restricted deletion of ATGL as well as those treated with atglistatin [13], an ATGL inhibitor that selectively targets ATGL in adipose tissue, but not in the heart, displayed reduced body weight and visceral adiposity. These metabolic benefits were associated with attenuated cardiac hypertrophy and, most importantly, improved diastolic dysfunction, the hallmark of HFpEF. Notably, mice with ATGL-deficient adipocytes were protected from diet-induced obesity and HFpEF, despite normal food intake. However, atglistatin reduced food/calorie intake in HFpEF mice by 31%, thereby inducing a state of moderate caloric restriction. Thus, to determine whether the effects of atglistatin stem solely from its impact on food/calorie intake, we matched the atglistatin-induced reduction of food intake in an independent group of calorically-restricted HFpEF mice. In doing so, we found that caloric restriction alone is sufficient to improve insulin and glucose homeostasis as well as reduce both cardiac hypertrophy and diastolic dysfunction. However, caloric restriction did not entirely recapitulate the benefits of atglistatin neither on cardiac function nor on visceral adiposity, despite similar body weight loss in both groups. Indeed, atglistatin was more effective in improving diastolic dysfunction and reducing visceral adiposity than caloric restriction. Collectively, these findings demonstrate the superior therapeutic efficacy of selectively targeting adipose tissue lipolysis compared with weight loss alone in mitigating obesity-related HFpEF (Fig. 1).

## Adipose tissue lipolysis inhibition interrupts a maladaptive fat-heart crosstalk in HFpEF

The cardiometabolic benefits of atglistatin and caloric restriction were associated with decreased levels of HFpEF-relevant cytokines, including tumor necrosis factor-alpha (TNF-α), interleukin-6 (IL-6), and interleukin-1beta (IL-1β) in adipose tissue of HFpEF mice. Notably, atglistatin conferred stronger anti-inflammatory effects than caloric restriction, especially on IL-1β, reflecting its superior capacity for cardioprotection against HFpEF. Mechanistically, adipocyte ATGL inhibition likely ameliorates cardiac inflammation through reducing galectin-3 secretion from adipose tissue [14]. However, other studies have also shown that the anti-inflammatory effects of ATGL suppression are associated with the activation of peroxisome proliferator-activated receptor (PPAR) signalling in adipocytes. Specifically, ATGL-deficient adipocytes have reduced levels of PPAR-α-target genes encoding pro-inflammatory cytokines [15]. Along the same line, a recent study showed that atglistatin-treated mice with metabolic dysfunction-associated steatohepatitis displayed significantly increased gene expression of the peroxisome proliferator-activated receptor gamma 2 (Pparg2) in adipose tissue [16], coinciding with reduced mRNA expression of proinflammatory cytokines. However, these mechanistic links between ATGL inhibition and IL-1β suppression remain to validated in the context of HFpEF.

Irrespectively, administration of IL-1β-neutralizing antibodies revealed that IL-1β inhibition alone improves HFpEF, although it failed to exhibit additional effects on diastolic dysfunction beyond those of atglistatin. These findings suggest that IL-1β drives a maladaptive fat-heart crosstalk in HFpEF. However, future studies need to determine whether IL-1β overexpression or administration diminishes the protective effects of atglistatin, thereby providing direct evidence that the atglistatin-mediated improvement in diastolic dysfunction is primarily dependent on IL-1β inhibition. This approach will also answer the question as to whether the benefits of atglistatin are driven by its potent anti-obesity effect or are rather due to the inhibition of IL-1β. To address these knowledge gaps, at least theoretically, we performed an analysis of covariance (ANCOVA), to assess whether atglistatin would still exert beneficial effects on diastolic function, regardless of body weight or visceral body fat mass change. Indeed, ANCOVA analysis, adjusting for body weight, showed that atglistatin still significantly improves diastolic dysfunction to a greater extent than caloric restriction alone. Additional ANCOVA analyses adjusting for either visceral adiposity mass or IL-1β confirmed that while both interventions significantly improved diastolic dysfunction, atglistatin remained superior to caloric restriction. Collectively, this hypothetical framework suggests that neither lower visceral adipose tissue mass nor reduced inflammation alone can fully explain the benefits of atglistatin, implying that rather the integration of several mechanisms drives these positive effects. However, and in contrast to atglistatin, caloric restriction appears to require body weight loss for its cardioprotective benefits because adjusting for body weight abolished the beneficial effect of caloric restriction on diastolic dysfunction. This distinction between atglistatin and caloric restriction is likely due to the fact that caloric restriction broadly modulates systemic metabolism across organs (eg, skeletal muscle, heart, adipose tissue), whereas atglistatin selectively targets adipose tissue metabolism. Furthermore, unlike caloric restriction, which lowers body weight through *activating* lipolysis, atglistatin exerts its effects by *inhibiting* lipolysis. Indeed, although calorically restricted-HFpEF mice and those receiving atglistatin display similar body weight reduction, visceral adiposity is significantly lower in atglistatin-treated mice, indicating depot-specific remodelling of adipose tissue. In this regard, future studies are warranted to test whether the cardioprotective effects of atglistatin also involve the reduction of epicardial adipose tissue, which has been also implicated in mediating deleterious effects of obesity and inflammation on the heart [17].

Notably, atglistatin exclusively accumulates in brown and white adipose tissue as well as the liver due to its highly hydrophobic structure [13]. As such, atglistatin is barely detectable in the heart, indicating that its cardiac effects are mediated by its extracardiac actions given that atglistatin does not inhibit cardiac ATGL but selectively inhibits lipolysis primarily in adipose tissue [18].

## Adipose tissue-specific ATGL inhibition: a potential alternative to body weight-lowering treatments of cardiometabolic HFpEF

From a clinical and translational perspective, the implementation of caloric restriction on a population level is challenging due to poor adherence. Furthermore, caloric restriction produces potential adverse effects, such as increased hunger, loss of lean mass, delayed wound healing, low bone mineral density, and impaired immunity [9]. By contrast, ATGL inhibition offers a promising pharmacological strategy that specifically and safely reduces visceral adiposity, while minimizing the risk of side effects. Thus, although fasting and caloric restriction do have benefits [5, 19], the specificity and practical advantages of targeting adipose tissue makes ATGL inhibitors a more feasible and potentially superior therapeutic strategy for managing obesity-related HFpEF. The anti-obesity effect is also relevant for GLP-1RAs and SGLT2 inhibitors, which demonstrated efficacy in HFpEF. However, it is important to note that these emerging therapies do not act directly or specifically on adipose tissue and, thus, through mechanisms that differ substantially from ATGL inhibition, which directly limits the release of non-esterified fatty acids from adipose tissue. This distinction is crucial, as lipotoxicity and adipose tissue-driven inflammation are central contributors to obesity-related HFpEF pathogenesis.

GLP-1RAs mimic caloric restriction by suppressing appetite and reducing food intake through central mechanisms, while SGLT2 inhibitors act on the kidney to prevent glucose reabsorption and induce osmotic diuresis. Moreover, despite their benefits, both therapies come with some side effects. On the one hand, GLP-1RAs are associated with skeletal muscle mass loss, which is particularly concerning in elderly patients at risk of sarcopenia. On the other hand, SGLT2 inhibitors can increase the risk of genital or urinary tract infections. By specifically targeting adipose tissue lipolysis, ATGL inhibition may avoid these potential side effects, and may offer an alternative in intolerant patients or those at high risk of these adverse effects, including elderly HFpEF patients with sarcopenic obesity.

## Concluding remarks

It is important to note that this study has some limitations. For instance, differences exist between mice and humans regarding white adipose tissue and lipid profiles [20]. Thus, future studies using additional animal models that also mimic human circulating lipoprotein profile that is dominated by very low density lipoproteins (VLDL)/low density lipoproteins (LDL) will further enhance the translational relevance. For this, expanding animal testing to *Ldlr*^*−/−*^ aged mice [21] subjected to a high-fat diet and angiotensin-II might be worth considering because their plasma lipids are confined to VLDL/LDL particles. Another limitation is that atglistatin acts on rodent ATGL, but does not target human ATGL, making mouse-to-human translation challenging. However, limited clinical applicability of atglistatin can be overcome with the novel small-molecule inhibitor of human ATGL, NG-497 [22], opening a new therapeutic perspective to validate the preclinical findings in patients with HFpEF. Accordingly, future clinical studies are needed to determine long-term safety profile and tolerability of ATGL inhibition in obese patients with HFpEF.

Notwithstanding these limitations, our findings suggest that adipocentric interventions inhibiting lipolysis specifically in adipose tissue and related inflammation interrupt the maladaptive fat-heart crosstalk, and effectively attenuate cardiac remodelling and dysfunction in obesity-related HFpEF. We propose that directly targeting intracellular fatty acids mobilization in adipose tissue (ie, lipolysis) could become a novel and effective approach for treating obesity-related HFpEF. Thus, inhibiting lipolysis specifically in adipose tissue by newly developed selective inhibitors of human ATGL [22], or stimulating fatty acid oxidation by other metabolic therapies that mimic caloric restriction [23], such as NAD^+^ precursors (eg, nicotinamide) [24, 25], might be considered as adjunctive or standalone therapies for obese patients with HFpEF, who are intolerant or at higher risk of adverse effects from currently available pharmacotherapies.

## Data Availability

No datasets were generated or analysed during the current study.
